# A conceptual analysis of SBIRT implementation alongside the continuum of PrEP awareness: domains of fit and feasibility

**DOI:** 10.3389/fpubh.2023.1310388

**Published:** 2024-01-08

**Authors:** Lesley M. Harris, Jelani C. Kerr, Blake D. Skidmore, Smita Ghare, Andrea Reyes-Vega, Vania Remenik-Zarauz, Harideep Samanapally, Rana Usman Anwar, Rishikesh Rijal, Kendall Bryant, Martin T. Hall, Shirish Barve

**Affiliations:** ^1^Kent School of Social Work & Family Science, University of Louisville, Louisville, KY, United States; ^2^Department of Health Promotion and Behavioral Sciences, University of Louisville, Louisville, KY, United States; ^3^School of Medicine, University of Louisville, Louisville, KY, United States; ^4^HIV/AIDS Research, National Institute on Alcohol Abuse and Alcoholism (NIAAA), Bethesda, MD, United States

**Keywords:** PrEP (pre-exposure prophylaxis), human immunodeficiency virus (HIV), HIV prevention and care, SBIRT alcohol, SBIRT implementation, transtheoretical model of change, motivational intervention/interviewing, care bundle approach

## Abstract

Screening, Brief Intervention, and Referral to Treatment (SBIRT) is a supplementary intervention that can be incorporated into the Pre-Exposure Prophylaxis (PrEP) Care Continuum, complementing initiatives and endeavors focused on Human Immunodeficiency Virus (HIV) prevention in clinical care and community-based work. Referencing the Transtheoretical Model of Change and the PrEP Awareness Continuum, this conceptual analysis highlights how SBIRT amplifies ongoing HIV prevention initiatives and presents a distinct chance to address identified gaps. SBIRT's mechanisms show promise of fit and feasibility through (a) implementing universal Screening (S), (b) administering a Brief Intervention (BI) grounded in motivational interviewing aimed at assisting individuals in recognizing the significance of PrEP in their lives, (c) providing an affirming and supportive Referral to Treatment (RT) to access clinical PrEP care, and (d) employing client-centered and destigmatized approaches. SBIRT is uniquely positioned to help address the complex challenges facing PrEP awareness and initiation efforts. Adapting the SBIRT model to integrate and amplify HIV prevention efforts merits further examination.

## Introduction

Pre-Exposure Prophylaxis (PrEP) is a once-daily oral medication currently available by prescription that is highly effective at preventing Human Immunodeficiency Virus (HIV). It reduces the risk of getting HIV from sex by about 99% and from injection drug use by at least 74% (1). A long-acting injectable PrEP option demonstrating greater effectiveness than oral PrEP was approved by the Food and Drug Administration in 2021 and has also become widely available (2). Localities with more PrEP use demonstrate lower HIV incidence. Although frequently marketed to populations historically viewed as more vulnerable to contracting HIV—specifically, men who have sex with men—PrEP can be used by anyone not currently diagnosed with HIV to prevent transmission.

Screening, Brief Intervention, and Referral to Treatment (SBIRT) is a comprehensive, evidence-based, integrated public health approach for early intervention and treatment. Originally designed as a model to engage persons with substance use disorders and those at risk of developing these disorders, the model begins with universal Screening (S) for risk and then uses Brief Intervention (BI) to increase individuals' awareness of risk and the benefits of behavior change. It culminates in Referral to Treatment (RT) for those at moderate to high risk. It has been adapted to address various health concerns, implemented in multiple settings, and shown to be effective (3–9).

According to CDC reports, there are several overarching challenges in HIV prevention, including issues related to awareness, treatment, equitable access to information, limited resources, stigma, complacency, and poverty (10). Studies on the challenges of PrEP awareness, initiation, and adherence highlight multiple factors, including stigma, medical mistrust, substance misuse, inequitable access and promotion, and high costs (11–17). Research examining what hinders an individual's movement along the PrEP Care Continuum has identified inadequate knowledge regarding how PrEP works, failure to translate PrEP's potential benefit to persons at higher risk of contracting HIV, and inaccurate information about PrEP limiting its initiation among populations experiencing disproportionate risk (18–20).

Current efforts to increase PrEP awareness and initiation are broad and have generated positive impacts. These include educational awareness campaigns, community outreach, conducting comprehensive risk assessments, providing tailored counseling and support to patients, increasing access to telehealth and digital interventions/services, and offering integrated and coordinated efforts to engage multiple prevention strategies (21–23). Together, these methods are proven to enhance the uptake and effectiveness of PrEP and highlight the need for combination approaches to HIV prevention (24).

In this conceptual analysis, we synthesize how SBIRT complements ongoing HIV prevention efforts, specifically the PrEP Care Continuum initiatives. The SBIRT model amplifies existing efforts, and its processes integrate screening and referrals and expedite PrEP-eligible individuals' movement from awareness to uptake, as conceptualized by the PrEP Care Continuum, through brief intervention.

## PrEP implementation aspects

### PrEP awareness and accessibility

Educational campaigns, healthcare provider training, and community outreach initiatives have played crucial roles in disseminating information about the benefits of PrEP. These awareness efforts aim to engage those at high risk of HIV infection and healthcare professionals who can facilitate the provision of PrEP (24, 25). In addition, policy changes and collaborations with pharmaceutical companies have helped reduce financial barriers, making PrEP more accessible to a broader range of individuals (26).

### Comprehensive risk assessment

One of the critical aspects of PrEP implementation is the development of comprehensive risk assessment strategies. Healthcare providers are encouraged to engage patients in open discussions about their sexual behaviors, substance use, and other risk factors (27). This approach ensures that PrEP is prescribed to those who genuinely need it, optimizing the impact of this medication on preventing new HIV infections (28).

### Tailored counseling and support

PrEP programs are increasingly integrating tailored and customized counseling and support services. These services provide patients with information about proper medication adherence, potential side effects, and the importance of regular medical check-ups (29, 30). By addressing concerns and providing ongoing guidance, healthcare providers and support staff contribute to higher adherence rates and the overall success of PrEP (31, 32). Sometimes, these counseling and support services use Motivational Interviewing (MI) (33) skills and approaches that have demonstrated MI's positive impact on HIV prevention, PrEP awareness, and initiation efforts (34).

MI posits that an individual's commitment to a behavior change can be enhanced by exploring the reason for the change and facilitating a conversation that allows the client to resolve any ambivalence about behavior change. In other words, it is “a collaborative conversational style for strengthening a person's motivation and commitment to change” (33, p. 12). Practitioners who use MI skills guide patients through exploring their wants or values, their ambivalence about making change, identifying a focus, and acting to attain their goals. This process is rooted in the goals and values of the client, which is a critical aspect of MI fidelity. In this way, MI contrasts with behaviors like confrontation (e.g., lecturing, shaming, coaxing, arguing) and persuasion (e.g., being overly directive with the client or offering unsolicited advice or advice without permission) (33). The impact of MI has demonstrated efficacy when used alone and in combination with other interventions (33, 35–37).

### Telehealth and digital interventions

The rise of telehealth and digital interventions within the COVID-19 era (38) has introduced new PrEP delivery and support avenues, including the emergence of artificial intelligence (AI) and machine learning (ML) (39). Remote consultations and online platforms allow individuals to access PrEP-related information, consultations, and prescription services using telehealth and other digital platforms. Service delivery systems such as Project ECHO and tele-mentoring effectively support patient and provider education and prescribing efforts. This approach is particularly beneficial for reaching individuals in rural or underserved areas and addressing geographical barriers to PrEP accessibility (40).

### Combination prevention strategies and complementary services

PrEP stands as a cornerstone of HIV prevention; however, it is increasingly being integrated into combination prevention strategies. These strategies combine methods such as condom use, regular testing for sexually transmitted infections, and harm reduction approaches for substance use (24). This comprehensive approach acknowledges that individuals have diverse needs and preferences, ensuring multiple layers of protection are in place (41, 42).

Existing HIV prevention efforts support access to and engagement in the PrEP Care Continuum, yet participation in the continuum remains low and higher participation is needed to prevent HIV. The progress in raising awareness, enhancing accessibility, and tailoring support services among persons at disproportionate risk has not translated into widespread PrEP usage. It is estimated that fewer than 20% of those eligible are adopting PrEP (43). Continuous efforts are needed to reduce stigma further, expand access, and increase initiation for those who can benefit the most. Without intervention, nearly 400,000 more Americans will be newly diagnosed over 10 years despite the availability of tools to prevent transmissions (44). The remaining gaps in care illuminate opportunities to combine PrEP with other prevention methods and embrace innovative approaches to increase awareness and initiation with more broad-reaching, patient-centered approaches and mechanisms designed to increase movement along the PrEP Care Continuum for eligible people.

## The transtheoretical model

The transtheoretical model (TTM) offers insights into relationships between PrEP awareness, initiation, and adherence. TTM has been applied in other aspects of HIV prevention (45–47), and it helps to answer questions about how an individual moves along the continuum. Within the TTM model, change is seen as a process through which individuals require different forms of support. These changes involve progress through six stages: pre-contemplation, contemplation, preparation, action, maintenance, and termination (48). In the first stage, individuals are unaware of a problem or lack the intention to take action to change. They then progress to a more critical awareness of a problem and begin preparing to take action. Behavior change occurs in the action stage, after which individuals work to maintain the positive change they have undertaken. Several interventions (i.e., educational campaigns to raise awareness, brief interventions to contemplate the pros and cons, and support services to assist with maintenance) are applied within each stage. TTM helps interventionists conceptualize change as a temporal process rather than an event. Interventions can then meet patients where they are and expedite their movement along the continuum. Examples include consciousness raising, self-evaluation, decisional balance, self-liberation (48), or, when situated within PrEP care, raising awareness of the medication, self-evaluating HIV risk, and initiating this highly effective medication.

## The continuum of PrEP awareness

To conceptualize an individual's PrEP usage, HIV prevention scholars have developed progressive, theoretical footholds called the Continuum of PrEP Awareness, documenting three phases (43). The initial phase involves an essential awareness of PrEP and an understanding that it can prevent HIV infection. The next phase expands awareness to include knowledge that PrEP is a daily prescription medication requiring ongoing monitoring. In the advanced phase, awareness encompasses the perception of PrEP as personally beneficial or, if not applicable personally, recognizing its value for others. For many, a passing interest in PrEP arises after initial exposure to the concept. However, this interest often needs more prompting and proactive steps toward seeking PrEP. In other words, awareness does not always lead to initiation, but awareness is a prerequisite to the possibility.

**Figure 1:**
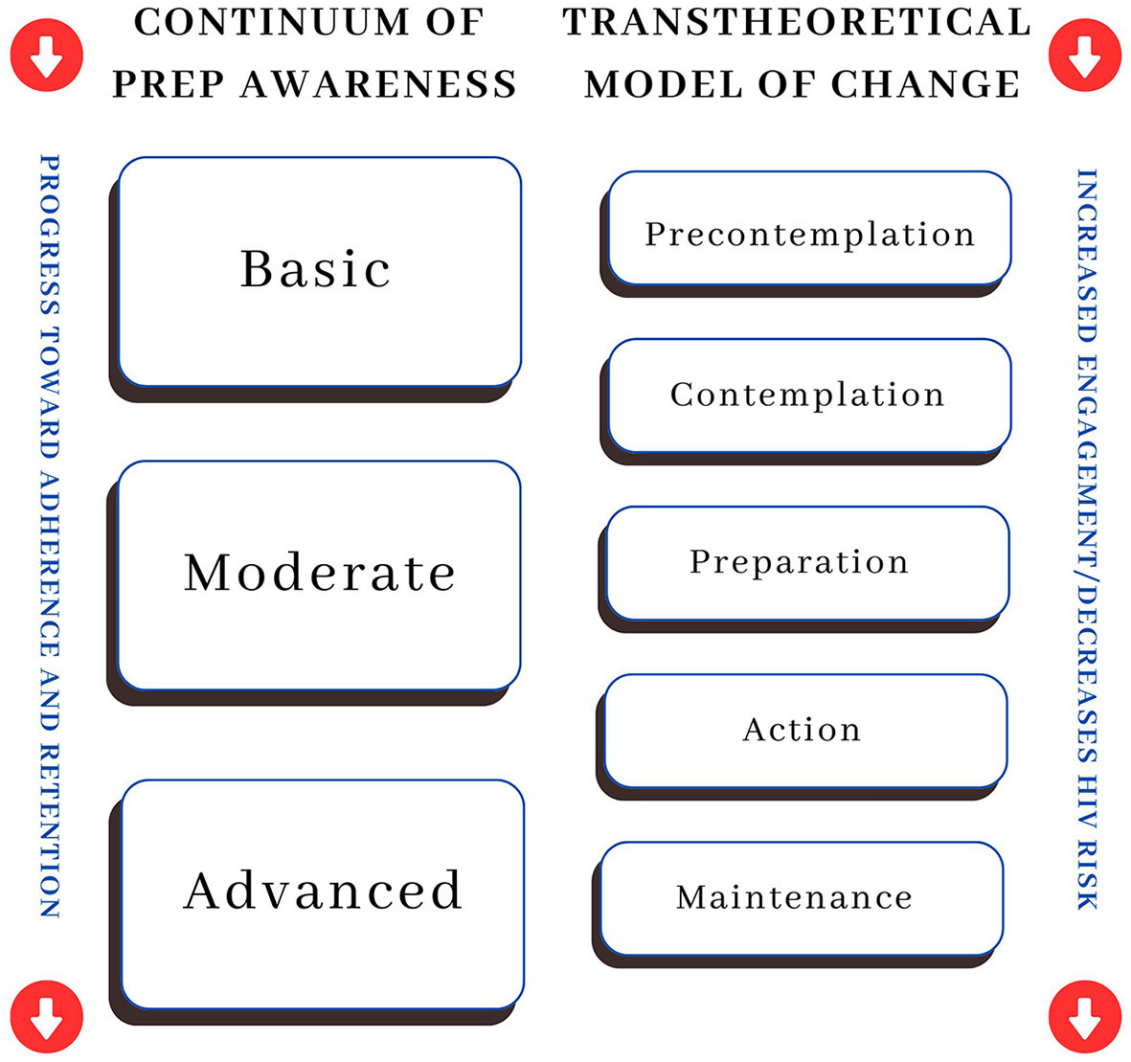
Integrating the continuum of PrEP awareness with the transtheoretical model of change.

This framing aligns with the recognized understanding of health behavior change outlined in the TTM. The Continuum of PrEP Awareness moves a person toward adherence and retention by beginning with the knowledge or a basic or an abstract understanding of PrEP. This process begins to bridge the gap between pre-contemplation and contemplation. As awareness and education grow, the person moves to the moderate stage, which includes an expanded knowledge of PrEP, including facts about and the requirements for taking the medication. This process aligns with the preparation stage of TTM. Lastly, the final stage is characterized by an advanced or complete understanding of logistics and potential benefits, which moves the person to uptake (or action, as described in TTM). Once engaged with PrEP, the focus shifts to adherence and retention, aligning with the TTM maintenance concept. Figure 1 represents the integration of the Continuum of PrEP Awareness with the TTM, highlighting how the movement from basic to advanced PrEP Awareness mirrors movement within the TTM from pre-contemplation to maintenance. Regression in the opposite direction of TTM steps can create a risk of PrEP disengagement and increase HIV risk.

Healthcare providers and community-based workers frequently play a crucial role in guiding patients through these awareness phases. In various instances, provider messages have made patients consider themselves suitable candidates for PrEP for the first time (14). However, the shift from awareness to talking with a healthcare provider about PrEP is rife with challenges, such as fear of PrEP, the burden of medication, stigma, lack of knowledgeable healthcare providers, mistrust of the healthcare system, and providers who fail even to discuss PrEP with eligible persons (39, 49, 50). Community-based workers are also at the frontline of educational campaigns, HIV testing, community outreach activities, and more. However, current strategies often need more systematic coordination or integration with one another. Additionally, providers need more tools to engage individuals at each TTM stage of change because, as Koester et al. (43) notes, awareness of PrEP only sometimes leads to opting to use it.

## SBIRT and PrEP care

SBIRT offers a unique opportunity to augment existing PrEP engagement efforts, first by integrating and amplifying several components of HIV prevention work and second by offering mechanisms to engage persons eligible for PrEP at each stage of the TTM, thus moving patients along the PrEP awareness continuum.

SBIRT effectively integrates awareness and accessibility efforts, comprehensive risk assessment, tailored counseling and support, combination prevention strategies, and complementary services. It begins through universal screening (S) designed to offer comprehensive *risk assessment* that invites open dialogue about the person's HIV risk as well as to offer *education and awareness* of PrEP's utility. The model then utilizes a brief intervention (BI) from MI techniques and a *tailored counseling approach*. This step operationalizes behavioral change by strengthening a patient's motivation and commitment to change, deeply exploring how PrEP may benefit them, facilitating their decisional balance process, and developing a plan for behavior change (which may include a referral to treatment). Lastly, the model emphasizes proactive referral to treatment, whether that is PrEP initiation, other HIV prevention resources, or *complementary services* that will support PrEP adherence. If the patient is high-risk and interested in getting help, they are referred to a more intensive treatment program. The implementation of the model is flexible and emphasizes the importance of cultural sensitivity and competence (51–53).

SBIRT draws its theory of change directly from the TTM and thus begins with tools to assist a patient in moving from pre-contemplation (or low awareness) to action (or advanced awareness). In addition, the model is grounded in patient-centered approaches that emphasize autonomy, collaboration, partnership, and compassion and uses methods to help individuals navigate from awareness of a problem to behavior change, or “initiation.” As noted above, MI has demonstrated efficacy when used alone and combined with other interventions like SBIRT. SBIRT is consistent with community-level outreach because it requires providers to engage people in conversations about moving through the awareness phases. In addition, the “spirit” of SBIRT fits PrEP and the community's long struggle with stigma. SBIRT's MI mechanisms include non-judgment, no assumptions, and are direct and motivating. These facets are necessary to reduce stigma and engage patients in PrEP. Below, we outline the fit and feasibility of combining SBIRT and the Continuum of PrEP Awareness.

## Fit and feasibility of combining SBIRT and the continuum of PrEP awareness

**Figure 2:**
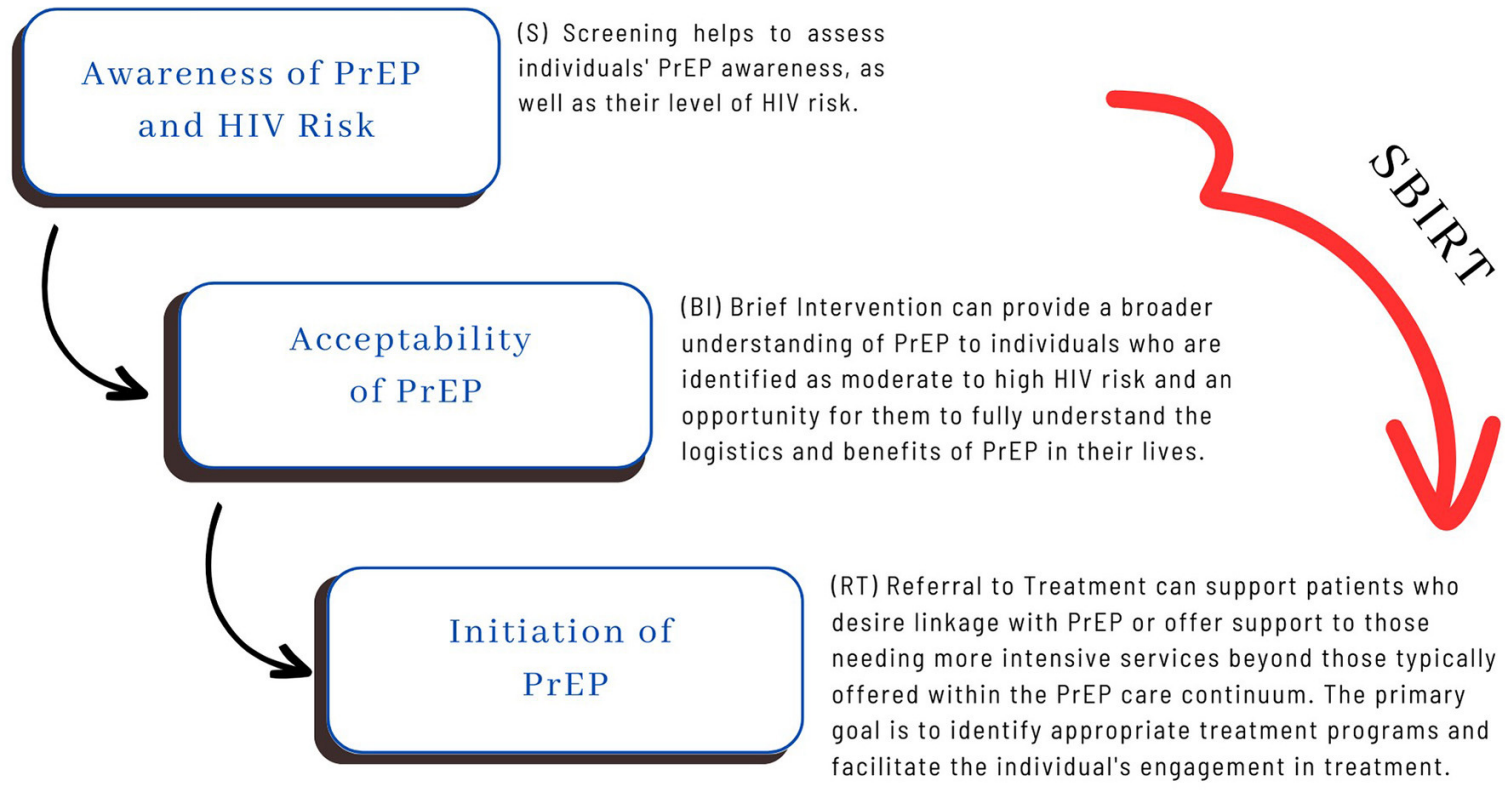
Integrating the continuum of PrEP awareness with SBIRT.

SBIRT may offer numerous advantages by mitigating HIV risk and assisting individuals engaged in risky behaviors with becoming better informed and seeking treatment. Furthermore, SBIRT empowers counselors, clinicians, and community health workers to identify and engage with patients who may not actively seek help, motivating and guiding them to understand PrEP, recognize HIV risk factors, and make healthier lifestyle choices proactively. Moreover, SBIRT seamlessly aligns with the PrEP Awareness Continuum as it can be effectively deployed across various settings dedicated to HIV prevention (See Figure 2). These settings include community-based organizations, outreach teams, mobile testing units, addiction health services, carceral settings, local health departments, faith-based organizations, youth-serving organizations, and schools. When the focus is on addressing HIV risk, implementing SBIRT in these contexts yields multiple benefits. It enables workers to screen individuals for high-risk behaviors, offers interventions geared toward improving sexual health, addresses high-risk sexual behaviors, provides education and early interventions, conducts initial assessments, assists patients in recognizing their HIV risk, and offers brief interventions and referrals to treatment.

Given the complexities within HIV care and the importance of interventions capable of reaching diverse and stigmatized communities, the SBIRT model is an essential tool in HIV prevention work. SBIRT integrates essential but discrete interventions into one cohesive model. It also offers a theoretical framework well-tailored to populations facing stigmatization or marginalization. Due to SBIRT's usability and proven impact on patient outcomes, it is uniquely positioned to help address challenges related to HIV prevention and PrEP awareness and initiation.

Koester's Continuum of PrEP Awareness (43) posits that optimizing PrEP uptake necessitates a recognition of PrEP's potential personal benefit. Information alone is insufficient for PrEP adoption, and intervention is needed for eligible persons to recognize heightened HIV risk and explore the benefits and drawbacks of PrEP use. SBIRT offers an integrated intervention model that supports these distinct processes. In the span of a 15 to 20-min engagement, persons are screened for risk, provided brief feedback on PrEP's benefits, invited to reflect on its advantages and disadvantages, and guided through the process of identifying potential help resources or services that might support their HIV care needs. This integrated process is facilitated in a non-judgmental and destigmatizing manner and culminates in a warm handoff to specialized or appropriate referrals. Below, we explain each step of SBIRT and describe its fit and feasibility with the PrEP awareness continuum.

### Screening

The initial (S) screening can be accomplished in 5–10 min. The subsequent intervention and treatment components indicated by the screening results are completed in significantly less time than traditional specialty care. The screening is universal; therefore, all patients are screened, and no one is left out of the intake process. Screening is also implemented every time the patient comes to the setting, which enables the provider to assess the patient's progress over time. The universal screening is completed with validated and standardized tools. It can include but is not limited to the HIV Risk-Taking Behavior Scale (54), PrEP Self-Efficacy Scale (55), PrEP Stigma Likert Scale (56), and tools to assess PrEP awareness and willingness to use PrEP (57).

### Brief intervention

A goal-oriented brief intervention (BI) targets one or more specific behaviors for patients identified by the screening as having moderate or high risk related to a health concern or behavior. The BI is an early intervention for individuals with moderate to high-risk thresholds. It is particularly suited for community-based settings because it aligns with patients' receptiveness to this form of intervention due to its pacing and engaging nature. BI also capitalizes on teachable moments and can motivate patients to seek additional support or treatment if necessary. Notably, it is a cost-effective approach compared to clinical care and offers flexibility by engaging a range of providers in varied settings. These interventions enhance patients' understanding and risk awareness and facilitate behavioral change. BI can be tailored to specific populations or settings and can serve as a stand-alone treatment or engage individuals needing more intensive care. Typically, BI is provided at the exact location where the screening occurred. The goal of BI, typically delivered in 1–5 sessions lasting from 5 min to an hour, is to educate patients and enhance their motivation to reduce high risk behaviors (53).

### Referral to treatment

Referral to treatment (RT) occurs when the provider needs to move beyond BI, directing patients needing a higher level of care to specialized treatment. The primary objectives of RT are to identify an appropriate treatment program and facilitate the patient's active involvement in the treatment. This process often involves coordination among various service providers. It necessitates a proactive and collaborative effort between SBIRT providers and specialists to ensure that referred patients can access and engage in appropriate care.

RT involves equipping community-based workers with essential resources for facilitating referrals and linkages to services, including PrEP treatment. This process necessitates that providers know about PrEP treatment options and maintain relationships with PrEP providers who are affirming and well-versed in providing care to populations who seek PrEP, inclusive of the LGBTQ+ community, men who have sex with men, persons who use injection drugs, and heterosexual individuals who have high-risk exposure.

To enhance patient engagement, SBIRT providers may employ motivational enhancement techniques to address any hesitations about treatment, arrange transportation for intake appointments, conduct follow-ups after appointments, and maintain communication with the specialty treatment provider. Establishing robust referral linkages and diligently tracking patient referrals are crucial. Providers must ensure that they have built up a network of LGBTQ+ and gender-affirming treatment providers specializing in substance use disorder recovery and mental health to optimize the continuum of SBIRT offerings. In certain states, gender-affirming care has been attacked by anti-trans legislation (58, 59), making it even more important to build a trusted referral network.

## Discussion

Over 40 years into the epidemic, HIV remains a significant public health crisis. PrEP has proven to be highly effective in altering the course of the epidemic; however, awareness, uptake, adherence, and retention remain challenging. PrEP engagement is complicated because many eligible persons are unaware of its availability and their HIV risk level (60). Others remain unconvinced that PrEP engagement would benefit them (61). Studies have shown that medical providers and HIV test counselors are credible messengers, and when they introduced patients to PrEP as an HIV prevention tool, patients were more likely to engage in uptake (43). Similar to SBIRT, providers often act as first responders and gateways to the introduction of PrEP information and offer advice that allows patients to view themselves as able and eligible to engage in care.

SBIRT offers a tool to increase PrEP initiation. Patients must (1) know they are at risk, (2) be aware of PrEP, (3) believe it is helpful or essential to them, and (4) know how to access it (43). Due to this multiple-step engagement process, a community-based approach, meeting people where they are on the PrEP awareness continuum and screening broadly, provides people with an awareness of their HIV risk, educates them about PrEP, and helps them explore its usefulness in their lives. From this point, accessing resources to engage in PrEP uptake, including a referral to a prescribing healthcare provider, is necessary. Community-based partnerships should exist with affirming and knowledgeable healthcare providers. Care needs to be delivered in the spirit of strengths-based empowerment, destigmatization, cultural responsiveness, compassion, and non-judgement and should be offered in conjunction with combination prevention strategies and complementary services.

In combating the HIV epidemic, it is imperative to implement effective programs and explore innovative approaches. Like SBIRT, care bundling is another useful strategy offering consolidation of services to enhance effectiveness by generating a synergy of efforts (62). In HIV prevention, bundling provides an advantage in delivering greater value at a reduced cost. This approach allows the simultaneous addressing of multiple risk behaviors, leveraging synergies for impact. The bundling of HIV prevention efforts creates an opportunity to reach individuals who are disproportionately impacted and may be reluctant to seek care (63), thereby eliminating barriers to preventive measures like PrEP. Similar to SBIRT, HIV prevention services have the potential to be consolidated or bundled within organizations possessing clinical capacity (such as emergency rooms, drug treatment centers, prisons, and shelters) as well as those lacking clinical facilities (including faith-based organizations, beauty salons, bars, and areas associated with drug and sex trade). Consequently, bundling opens novel avenues for outreach, especially among those at high risk or those whose risk may be underestimated (64). In this way, SBIRT and care bundling strategies create an opportunity to address multiple risk behaviors simultaneously. Future research on the application of the SBIRT-PrEP model with small populations would be informative and, like care bundles, might have a positive impact on treatment effectiveness and costs when compared to the current HIV prevention approaches.

PrEP is often hailed as a groundbreaking advancement in HIV prevention, potentially elevating the fundamental human rights of individuals by promoting enhanced standards of sexual health (65). Our conceptual analysis aimed to delve into the mechanisms underlying how individuals can acquire knowledge and make informed choices about PrEP while integrating SBIRT to leverage this awareness continuum. This improved understanding of SBIRT implementation alongside the Continuum of PrEP Awareness carries significant implications for future program implementation, outreach initiatives, and community education endeavors.

## Author contributions

LH: Conceptualization, Funding acquisition, Visualization, Writing—original draft, Writing—review & editing, Methodology, Supervision. JK: Conceptualization, Visualization, Writing—original draft, Writing—review & editing, Funding acquisition, Methodology, Supervision. BS: Conceptualization, Visualization, Writing—original draft, Writing—review & editing. SG: Conceptualization, Funding acquisition, Project administration, Visualization, Writing—review & editing. AR-V: Conceptualization, Writing—review & editing, Project administration, Supervision. VR-Z: Conceptualization, Writing—review & editing. HS: Conceptualization, Writing—review & editing. RA: Conceptualization, Writing—review & editing. RR: Conceptualization, Writing—review & editing. KB: Conceptualization, Writing—review & editing. MH: Conceptualization, Funding acquisition, Visualization, Writing—original draft, Writing—review & editing. SB: Conceptualization, Funding acquisition, Project administration, Supervision, Visualization, Writing—review & editing.

## References

[1] Centers for Disease Control and Prevention. PrEP Effectiveness. (2022). Available online at: https://www.cdc.gov/hiv/basics/prep/prep-effectiveness.html (accessed June 6, 2022).

[2] Landovitz RJ Donnell D Clement ME Hanscom B Cottle L Coelho L. Cabotegravir for HIV prevention in cisgender men and transgender women. N Engl J Med. (2021) 385:595–608. 10.1056/NEJMoa2101016 34379922 PMC8448593

[3] Bernstein E Bernstein JA Stein JB Saitz R SBIRT. in emergency care settings: Are we ready to take it to scale? J Acad Emerg Med. (2009) 16:1072–7. 10.1111/j.1553-2712.2009.00549.x 20053225

[4] Bruguera P Barrio P Manthey J Oliveras C López-Pelayo H Nuño L. Mid and long-term effects of a SBIRT program for at-risk drinkers attending to an emergency department. Follow-up results from a randomized controlled trial. Eur J Emerg Med. (2021) 28:373–9. 10.1097/MEJ.0000000000000810 33709997

[5] Castater C Raney E Nguyen J Reed KK Thompson AN Greene WR. Screening, Brief Intervention, and Referral to Treatment to prevent post-traumatic stress disorder after gunshot wounds. Am Surg. (2022) 88:2215–7. 10.1177/00031348221091955 35503305

[6] Gilbert L Shaw SA Goddard-Eckrich D Chang M Rowe J McCrimmon T. Project WINGS (women initiating new goals of safety): a randomised controlled trial of a screening, brief intervention and referral to treatment (SBIRT) service to identify and address intimate partner violence victimisation among substance-using women receiving community supervision. Crim Behav Mental Health. (2015) 25:314–29. 10.1002/cbm.1979 26482019

[7] Gryczynski J Mitchell SG Peterson TR Gonzales A Moseley A Schwartz RP. The relationship between services delivered and substance use outcomes in new mexico's screening, brief intervention, referral and treatment (SBIRT) Initiative. Drug Alcohol Depend. (2011) 118:152–7. 10.1016/j.drugalcdep.2011.03.012 21482039 PMC3158968

[8] Madras BK Compton WM Avula D Stegbauer T Stein JB Clark HW. Screening, brief interventions, referral to treatment (SBIRT) for illicit drug and alcohol use at multiple healthcare sites: Comparison at intake and 6 months later. Drug Alcohol Depend. (2009) 99:280–95. 10.1016/j.drugalcdep.2008.08.003 18929451 PMC2760304

[9] Young MM Stevens A Galipeau J Pirie T Garritty C Singh K. Effectiveness of brief interventions as part of the screening, brief intervention and referral to treatment (SBIRT) model for reducing the nonmedical use of psychoactive substances: a systematic review. Syst Rev. (2014) 3:1–18. 10.1186/2046-4053-3-50 24887418 PMC4042132

[10] Centers for Disease Control and Prevention. HIV by Age: Prevention Challenges. (2022). Available online at: https://www.cdc.gov/hiv/group/age/prevention-challenges.html (accessed April 6, 2022)

[11] Ayangeakaa SD Kerr J Combs R Harris LM Sears J. Understanding influences on intention to use pre-exposure prophylaxis (PrEP) among African American young adults. J Racial Ethn Health Disparities. (2022) 10:899–910. 10.1007/s40615-022-01278-7 35290648

[12] Ayangeakaa SD Kerr J Combs RM Harris LM Sears JS Parker K. Sociocultural and structural influences on HIV pre-exposure prophylaxis (PrEP) engagement and uptake among African American Young adults. BMC Pub Health. (2023) 23:1427 10.1186/s12889-023-16273-8 37495954 PMC10369814

[13] Ching SZ Wong LP Said MAB Lim SH. Meta-synthesis of qualitative research of Pre-exposure Prophylaxis (PrEP) adherence among men who have sex with men (MSM). AIDS Educ Prev. (2020) 32:416–31. 10.1521/aeap.2020.32.5.416 33112675

[14] Kerr J Ayangeakaa S Combs R Harris L Sears J Northington T. Community-informed development of a campaign to increase HIV pre-exposure prophylaxis (PrEP) awareness among African-American young adults. J Racial Ethnic Health Dispar. (2021) 8:901–11. 10.1007/s40615-020-00848-x 32869211

[15] Kerr J Ayangeakaa S Bullock NA Burton K Combs R Harris L. Qualitative exploration of various stigmas impacting HIV pre-exposure prophylaxis (PrEP) uptake among African American young adults. Fam Commun Health. (2022) 45:218. 10.1097/FCH.0000000000000346 35985022 PMC10111377

[16] Shuper PA Varatharajan T Kinitz DJ Gesink D Joharchi N Bogoch II. Perceived influence of alcohol consumption, substance use, and mental health on PrEP adherence and condom use among PrEP-prescribed gay, bisexual, and other men-who-have-sex-with-men: a qualitative investigation. BMC Pub Health. (2022) 22:1875. 10.1186/s12889-022-14279-2 36207757 PMC9540691

[17] Wood S Gross R Shea JA Bauermeister JA Franklin J Petsis D. Barriers and facilitators of PrEP adherence for young men and transgender women of color. AIDS Behav. (2019) 23:2719–29. 10.1007/s10461-019-02502-y 30993479 PMC6790163

[18] Olansky E Mansergh G Pitts N Mimiaga MJ Denson DJ Landers S. PrEP awareness in the context of HIV/AIDS conspiracy beliefs among Black/African American and Hispanic/Latino MSM in three urban US cities. J Homosex. (2019) 67:833–43. 10.1080/00918369.2018.1557953 30633661

[19] Sevelius JM Deutsch MB Grant R. The future of PrEP among transgender women: the critical role of gender affirmation in research and clinical practices. J Int AIDS Soc. (2016) 19:1–7. 10.7448/IAS.19.7.21105 27760683 PMC5071750

[20] Sullivan PS Sanchez TH Zlotorzynska M Chandler CJ Sineath RC Kahle E. National trends in HIV pre-exposure prophylaxis awareness, willingness and use among United States men who have sex with men recruited online, 2013 through 2017. J Int AIDS Soc. (2020) 23:e25461. 10.1002/jia2.25461 32153119 PMC7062633

[21] Bonacci RA Smith DK Ojikutu BO. Toward greater pre-exposure prophylaxis equity: Increasing provision and uptake for Black and Hispanic/Latino individuals in the US. Am J Prev Med. (2021) 61:S60–72. 10.1016/j.amepre.2021.05.027 34686293 PMC8668046

[22] Garrison LE Haberer JE. Pre-exposure prophylaxis uptake, adherence, and persistence: a narrative review of interventions in the US. Am J Prev Med. (2021) 61:S73–86. 10.1016/j.amepre.2021.04.036 34686294

[23] Kudrati SZ Hayashi K Taggart T. Social media and PrEP: a systematic review of social media campaigns to increase PrEP awareness and uptake among young Black and Latinx MSM and women. AIDS Behav. (2021) 25:4225–34. 10.1007/s10461-021-03287-9 33939035 PMC8563493

[24] Kerr J Lelutiu-Weinberger C Nelson LE Turan JM Frye V Matthews DW. Addressing intersectional stigma in programs focused on ending the HIV epidemic. Am J Pub Health. (2022) 112:S362–6. 10.2105/AJPH.2021.306657 35763743 PMC9241451

[25] Storholm ED Ober AJ Mizel ML Matthews L Sargent M Todd I. Primary care providers' knowledge, attitudes, and beliefs about HIV Pre-exposure Prophylaxis (PrEP): Informing network-based interventions. AIDS Educ Prev. (2021) 33:325–44. 10.1521/aeap.2021.33.4.325 34370571 PMC8559721

[26] Tan DH Dashwood TM Wilton J Kroch A Gomes T Martins D. Trends in HIV pre-exposure prophylaxis uptake in Ontario, Canada, and impact of policy changes: A population-based analysis of projected pharmacy data (2015–2018). Can J Pub Health. (2021) 112:89–96. 10.17269/s41997-020-00332-3 32529552 PMC7851246

[27] Przybyla S LaValley S Vil NS. Health care provider perspectives on pre-exposure prophylaxis: a qualitative study. J Assoc Nurses AIDS Care. (2019) 30:630–8. 10.1097/JNC.0000000000000073 30958406

[28] Faryar KA Ancona RM Braun RS Brown JL Sickles RK Lyons MS. Estimated proportion of an urban academic emergency department patient population eligible for HIV preexposure prophylaxis. Am J Emerg Med. (2021) 48:198–202. 10.1016/j.ajem.2021.04.087 33975131

[29] Amico KR Barta W Konkle-Parker DJ Fisher JD Cornman DH Shuper PA. The information–motivation–behavioral skills model of ART adherence in a deep South HIV+ clinic sample. AIDS Behav. (2009) 13:66–75. 10.1007/s10461-007-9311-y 17876697 PMC3018830

[30] Amico KR Mansoor LE Corneli A Torjesen K van der Straten A. Adherence support approaches in biomedical HIV prevention trials: experiences, insights and future directions from four multisite prevention trials. AIDS Behav. (2013) 17:2143–55. 10.1007/s10461-013-0429-9 23435697 PMC3672509

[31] Desrosiers A Levy M Dright A Zumer M Jallah N Kuo I. A randomized controlled pilot study of a culturally-tailored counseling intervention to increase uptake of HIV pre-exposure prophylaxis among young Black men who have sex with men in Washington, DC. AIDS Behav. (2019) 23:105–15. 10.1007/s10461-018-2264-5 30171452 PMC6344254

[32] Rousseau E Julies RF Madubela N Kassim S. Novel platforms for biomedical HIV prevention delivery to key populations—Community mobile clinics, peer-supported, pharmacy-Led PrEP delivery, and the use of telemedicine. Curr HIV/AIDS Rep. (2021) 18:1–8. 10.1007/s11904-021-00578-7 34708316 PMC8549812

[33] Miller WR Rollnick S. Motivational Interviewing: Helping People Change. London: Guilford Press (2012).

[34] Dangerfield DT Davis G Pandian V Anderson JN. Using Motivational Interviewing to increase HIV PrEP initiation and adherence: a scoping review. Prev Sci. (2023) 18:1–11. 10.1007/s11121-023-01554-w PMC10575988 37249729

[35] Amrhein PC Miller WR Yahne CE Palmer M Fulcher L. Client commitment language during motivational interviewing predicts drug use outcomes. J Consult Clin Psychol. (2003) 71:862–78. 10.1037/0022-006X.71.5.862 14516235

[36] Magill M Gaume J Apodaca TR Walthers J Mastroleo NR Borsari B. The technical hypothesis of motivational interviewing: a meta-analysis of MI's key causal model. J Consult Clin Psychol. (2014) 82:973–83. 10.1037/a0036833 24841862 PMC4237697

[37] Magill M Apodaca TR Borsari B Gaume J Hoadley A Gordon REF. A meta-analysis of motivational interviewing process: Technical, relational, and conditional process models of change. J Consult Clin Psychol. (2018) 86:140–57. 10.1037/ccp0000250 29265832 PMC5958907

[38] Harris LM Marsh JC Khachikian T Serret V Kong Y Guerrero EG. What can we learn from COVID-19 to improve opioid treatment? Expert providers respond. J Subst Abuse Addict Treat. (2023) 154. 10.1016/j.josat.2023.209157 37652210 PMC10923184

[39] Marcus JL Sewell WC Balzer LB Krakower DS. Artificial intelligence and machine learning for HIV prevention: emerging approaches to ending the epidemic. Curr HIV/AIDS Rep. (2020) 17:171–9. 10.1007/s11904-020-00490-6 32347446 PMC7260108

[40] Wood BR Mann MS Martinez-Paz N Unruh KT Annese M Spach DH. Project ECHO: Telementoring to educate and support prescribing of HIV pre-exposure prophylaxis by community medical providers. Sex Health. (2018) 15:601–5. 10.1071/SH18062 30318034

[41] Pettifor A Nguyen NL Celum C Cowan FM Go V Hightow-Weidman L. Tailored combination prevention packages and PrEP for young key populations. J Int AIDS Soc. (2015) 18:19434. 10.7448/IAS.18.2.19434 25724507 PMC4344537

[42] Ravasi G Grinsztejn B Baruch R Guanira JV Luque R Cáceres CF. Towards a fair consideration of PrEP as part of combination HIV prevention in Latin America. J Int AIDS Soc. (2016) 19:7. 10.7448/IAS.19.7.21113 27760687 PMC5071748

[43] Koester KA Erguera XA Udoh I Kang Dufour MS Burack JH Myers JJ. Exploring the shift from HIV pre-exposure prophylaxis awareness to uptake among young gay and bisexual men. Front Pub Health. (2021) 9:677716. 10.3389/fpubh.2021.677716 34950622 PMC8688695

[44] HIV.gov. What Is Ending the HIV Epidemic in the U.S.? (2023). Available online at: https://www.hiv.gov/federal-response/ending-the-hiv-epidemic/overview/ (accessed September 20, 2023)

[45] Genberg BL Lee Y Rogers WH Willey C Wilson IB. Stages of change for adherence to antiretroviral medications. AIDS Patient Care STDs. (2013) 27:567–72. 10.1089/apc.2013.0126 24093810 PMC3791039

[46] Longmire-Avital B Golub SA Parsons JT. Self-reevaluation as a critical component in sustained viral load change for HIV+ adults with alcohol problems. Ann Behav Med. (2010) 40:176–83. 10.1007/s12160-010-9194-4 20668976 PMC2939147

[47] Parsons JT Golub SA Rosof E Holder C. Motivational interviewing and cognitive-behavioral intervention to improve HIV medication adherence among hazardous drinkers: a randomized controlled trial. J Acquir Immune Defic Syndr. (2007) 46:443–50. 10.1097/qai.0b013e318158a461 18077833 PMC2666542

[48] Prochaska JO Velicer WF. The transtheoretical model of health behavior change. Am J Health Promot. (1997) 12:38–48. 10.4278/0890-1171-12.1.38 10170434

[49] Bauermeister JA Meanley S Pingel E Soler JH Harper GW. PrEP awareness and perceived barriers among single young men who have sex with men in the United States. Curr HIV Res. (2013) 11:520–7. 10.2174/1570162X12666140129100411 24476355 PMC4152728

[50] García M Harris AL. PrEP awareness and decision-making for Latino MSM in San Antonio, Texas. PLoS ONE. (2017) 12:e0184014. 10.1371/journal.pone.0184014 28953905 PMC5617149

[51] Hargraves D White C Frederick R Cinibulk M Peters M Young A. Implementing SBIRT (screening, brief intervention and referral to treatment) in primary care: lessons learned from a multi-practice evaluation portfolio. Public Health Rev. (2017) 38:31. 10.1186/s40985-017-0077-0 29450101 PMC5809898

[52] Manuel JK Satre DD Tsoh J Moreno-John G Ramos JS McCance-Katz EF. Adapting screening, brief intervention, and referral to treatment for alcohol and drugs to culturally diverse clinical populations. J Addict Med. (2015) 9:343–51. 10.1097/ADM.0000000000000150 26428359 PMC4626638

[53] Substance Abuse and Mental Health Services Administration. Screening, Brief Intervention, and Referral to Treatment (SBIRT): A National Strategy to Prevent and Reduce Alcohol and Other Drug Problems. Washington, DC: U.S. Department of Health and Human Services (2014).

[54] Ward J Darke S Hall W. The HIV Risk-Taking Behaviour Scale (HRBS) Manual. Sydney, NSW: National Drug and Alcohol Research Centre, University of New South Wales (1990).

[55] Golub SA Starbuck L Fikslin R Gamarel KE. Psychometric evaluation and predictive validity of an adapted adherence self-efficacy scale for PrEP. AIDS Behav. (2023) 27:218–30. 10.1007/s10461-022-03758-7 35809144

[56] Klein H Washington TA. The pre-exposure prophylaxis (PrEP) stigma scale: preliminary findings from a pilot study. Int J Pub Health. (2019) 11:185–95. 10.1007/s10461-012-0313-z 32089789 PMC7034943

[57] Wilton J Kain T Fowler S Hart TA Grennan T Maxwell J. Use of an HIV-risk screening tool to identify optimal candidates for PrEP scale-up among men who have sex with men in Toronto, Canada: disconnect between objective and subjective HIV risk. J Int AIDS Soc. (2016) 19:20777. 10.7448/IAS.19.1.20777 27265490 PMC4911732

[58] Ducar D. Attacks on affirming health care require a unified response. J Psychosoc Nurs Ment Health Serv. (2023) 61:2–3. 10.3928/02793695-20230215-01 36881805

[59] Mallory C Chin MG Lee JC. Legal penalties for physicians providing gender affirming care. JAMA. (2023) 329:1821–2. 10.1001/jama.2023.8232 37200027

[60] Strauss BB Greene GJ Phillips G Bhatia R Madkins K Parsons JT. Exploring patterns of awareness and use of HIV pre-exposure prophylaxis among young men who have sex with men. AIDS Behav. (2017) 21:1288–98. 10.1007/s10461-016-1480-0 27401537 PMC5226918

[61] Holt M Murphy D Callander D Ellard J Rosengarten M Kippax S. HIV-negative and HIV-positive gay men's attitudes to medicines, HIV treatments and antiretroviral-based prevention. AIDS Behav. (2013) 17:2156–61. 23001412 10.1007/s10461-012-0313-z

[62] Ickovics JR. “Bundling” HIV prevention: integrating services to promote synergistic gain. Prev Med. (2008) 46:222–5. 10.1016/j.ypmed.2007.09.006 17964637 PMC2276879

[63] Grussing ED Pickard B Khalid A Smyth E Childs V. Implementation of a bundle to improve HIV testing during hospitalization for people who inject drugs. Implement Res Pract. (2023) 4:26334895231203410. 10.1177/26334895231203410 37936964 PMC10548809

[64] Eaton LA Huedo-Medina TB Kalichman SC Pellowski JA Sagherian MJ Warren M. Meta-analysis of single-session behavioral interventions to prevent sexually transmitted infections: implications for bundling prevention packages. Am J Public Health. (2012) 102:e34–44. 10.2105/AJPH.2012.300968 22994247 PMC3477958

[65] Calabrese SK Underhill K. How stigma surrounding the use of HIV preexposure prophylaxis undermines prevention and pleasure: a call to destigmatize “truvada whores”. Am J Pub Health. (2015) 105:1960–4. 10.2105/AJPH.2015.302816 26270298 PMC4566537

